# Exploring blood alterations in chronic kidney disease and haemodialysis using metabolomics

**DOI:** 10.1038/s41598-020-76524-1

**Published:** 2020-11-11

**Authors:** Yoric Gagnebin, David A. Jaques, Serge Rudaz, Sophie de Seigneux, Julien Boccard, Belén Ponte

**Affiliations:** 1 School of Pharmaceutical Sciences, University of Geneva, Geneva, Switzerland; 2 Institute of Pharmaceutical Sciences of Western Switzerland, University of Geneva, Geneva, Switzerland; 3 Service of Nephrology and Hypertension, Department of Medicine, Geneva University Hospitals, Rue Gabrielle-Perret-Gentil 4, 1205 Geneva, Switzerland; 4 Swiss Centre for Applied Human Toxicology, University of Basel, Basel, Switzerland

**Keywords:** Diagnostic markers, Chronic kidney disease, Haemodialysis, Metabolomics

## Abstract

Chronic kidney disease (CKD) is characterized by retention of uremic solutes. Compared to patients with non-dialysis dependent CKD, those requiring haemodialysis (HD) have increased morbidity and mortality. We wished to characterise metabolic patterns in CKD compared to HD patients using metabolomics. Prevalent non-HD CKD KDIGO stage 3b–4 and stage 5 HD outpatients were screened at a single tertiary hospital. Various liquid chromatography approaches hyphenated with mass spectrometry were used to identify 278 metabolites. Unsupervised and supervised data analyses were conducted to characterize metabolic patterns. 69 patients were included in the CKD group and 35 in the HD group. Unsupervised data analysis showed clear clustering of CKD, pre-dialysis (preHD) and post-dialysis (postHD) patients. Supervised data analysis revealed qualitative as well as quantitative differences in individual metabolites profiles between CKD, preHD and postHD states. An original metabolomics framework could discriminate between CKD stages and highlight HD effect based on 278 identified metabolites. Significant differences in metabolic patterns between CKD and HD patients were found overall as well as for specific metabolites. Those findings could explain clinical discrepancies between patients requiring HD and those with earlier stage of CKD.

## Introduction

Metabolomics is a systems biology approach aiming at identifying and quantifying metabolites in a given biological sample^1^. As metabolites can be seen as final products of physiological homeostasis, metabolomics complements other “omics” techniques in an attempt to phenotypically characterize an entire biological system^1,2^. The main analytical techniques used in chronic kidney disease (CKD) metabolomics are nuclear magnetic resonance spectroscopy (NMR) and mass spectrometry (MS) usually preceded by a separation technique such as liquid chromatography (LC–MS), gas chromatography (GC–MS) or capillary electrophoresis (CE-MS)^3^. Since no single approach can provide exhaustive metabolome coverage, new workflows combining different chromatographic separation modes have recently received strong interest^4^. Most CKD metabolomics studies relied on reversed phase liquid chromatography (RPLC), which offers effective separation and retention for relatively non-polar metabolites ^5,6^. The exclusive use of this method however might introduce bias towards lipophilic metabolites to the detriment of polar metabolites of high significance, such as amino acids^7^. Hydrophilic interaction chromatography (HILIC) has recently become increasingly popular to analyse polar metabolites and several studies showed its potential to enhance metabolome coverage and its viability as a CKD chromatographic separation mode^3,7,8^.

CKD is a worldwide health burden with an estimated global prevalence of 11% to 13% that is associated with an increased risk of all-cause and cardiovascular mortality^9,10^. Small molecules that accumulate in CKD and exert detrimental biological activity are termed uremic toxins and are thought to contribute to mortality^11^. Other characteristic features of CKD such as catabolism as well as disturbances in amino acid and lipid metabolism might also contribute to adverse outcomes^12–14^. As a consequence of CKD, over two million patients are treated with haemodialysis (HD) worldwide^15^. While HD is designed to restore solutes and volume homeostasis, mortality universally increases early after dialysis initiation and non-HD CKD patients maintain considerably lower overall mortality risk compared to patients requiring HD^16,17^. A global understanding of the impact of HD on the metabolic disarray characterising CKD is thus pivotal in order to fully characterise the clinical trajectories of those patients and potentially improve therapeutic strategies. However, while several metabolomics studies included HD patients, most did not analyse the impact of dialysis itself or did not describe CKD patients concomitantly^5,18–22^. As such, the impact of HD on metabolic profiles in CKD patients has not been thoroughly described.

In the present study, we present an original metabolomics workflow based on a combination of RPLC and HILIC separation modes in order to enhance metabolome coverage. We apply this strategy to the study of CKD as well as HD patients. Our main goal was to characterise the impact of HD on blood metabolic patterns, while considering CKD patients not requiring dialysis as a reference point. We hypothesized that HD patients would present substantial differences in metabolic profiles as compared to non-HD CKD patients, thereby potentially explaining clinical specificities encountered in daily practice.

## Materials and methods

### Participants’ selection

We performed an observational monocentric study at a tertiary hospital (Geneva Univerisity Hospitals, Geneva, Switzerland). Participants were prevalent non-HD CKD patients (CKD group) followed by nephrologists at the hospital outpatient clinic as well as CKD patients undergoing chronic in-hospital HD (HD group). The aim was to include at least 50 + /− 10 patients in the CKD group and 30 + /− 5 patients in the HD group. Inclusion criteria were: age ≥ 18 years, able to provide informed consent, CKD KDIGO stage 3b–4 (eGFR 44 —15 mL/min/1.73 m^2^) for the CKD group or stage 5 (< 15 mL/min/1.73 m^2^) currently on HD for the HD group. Patients in the HD group had to be on chronic HD for at least 3 months on a standard regimen of 4 h, 3 times per week. For samples processing reasons, we only included patients dialysed in the morning shift. Incapacity to give consent and pregnancy were the only exclusion criteria. Patients in the CKD group have been explored in a previous manuscript describing the detailed analytical protocol^23^.

### Dialysis characteristics

All participants in the HD group received post-dilution haemodiafiltration with high-flux filters. Dialysate solutions were standard with isonatremic sodium concentration, ionized calcium of 1.5 mmol/L, bicarbonate of 31 mmol/L and potassium of 3 mmol/L. The investigators did not interfere with dialysis prescription, and ultrafiltration was left to the clinician consideration.

### Sample preparation and analysis

Clinical and demographic variables were collected at inclusion. All plasma samples were collected at the same time in the morning in fasting patients. For patients in the HD group, samples were drawn before (preHD group) and after (postHD group) dialysis in each participant on the mid-week session. All samples were directly thawed, aliquoted and stored at − 80 °C. Samples were randomly analysed in 4 batches. A series of 15 quality control (QC) injections was carried out at the beginning of each batch for system conditioning. Solvent blanks were injected within each acquisition sequence to assess potential carryover effects, while standard mixtures were used to ensure mass accuracy. QC and diluted QC (dQC) samples were injected for data filtering, analytical variability evaluation and normalization (1 injection every 6 samples). Creatinine levels were measured locally according to clinical need, on the same day as the other samples for metabolomic measurements. Glomerular filtration rate was estimated (eGFR) using Chronic Kidney Disease Epidemiology Collaboration (CKD-EPI) formula^24^.

### Metabolomics analysis

A detailed description of the analytical protocol, which is briefly summarized here, was previously published^23^. Sample preparation included protein precipitation carried out using cold methanol spiked with isotopically labelled standards, and centrifugation. Chromatography was performed on a Waters H-Class Acquity UPLC system composed of a quaternary pump, a column manager and an FTN auto sampler (Waters Corporation, Milford, MA, USA). A Phenomenex Kinetex C18 column (150 × 2.1 mm, 1.7 µm), preceded by a SecurityGuard ULTRA pre-column, was used for RPLC separation. A Waters Acquity BEH Amide column (150 × 2.1 mm, 1.7 µm) preceded by a VanGuard pre-column was used for HILIC separation with amide-bonded stationary phase (aHILIC), while a Merck SeQuant Zic-pHILIC column (150 × 2.1 mm, 5 μm) and appropriate guard kit was applied for HILIC separation with polymeric zwitterionic stationary phase (ZICpHILIC). Samples were randomly analysed in 4 batches. QC and dQC samples were injected for data filtering, analytical variability evaluation and normalization (1 injection every 6 samples). The Waters H-Class Acquity UPLC system was coupled to a maXis 3G Q-TOF high-resolution MS (full sensitivity resolution of > 40,000) from Bruker (Bruker Daltonik GmbH, Bremen, Germany) with an electrospray ionization source working in positive (ESI+) or negative (ESI−) mode. Data between 50 and 1000 m/z were acquired in profile mode at a rate of 2 Hz.

### Data processing and analysis

Data processing was performed using Progenesis QI 2.3 (Nonlinear Dynamics, Waters, Newcastle upon Tyne, UK). Data filtering was carried out by applying a threshold of the dQC/QC ratio relative standard deviation (RSD) of 50% and a dQC/QC ratio between 0.2 and 0.8. QC-based LOESS regression was used for intra- and inter-batch normalization. Metabolite identifications with the highest level of confidence (i.e. level 1) were achieved using an in-house library containing experimental data from more than 900 authentic standards acquired in the same chromatographic conditions^25,26^. Level 1 annotation was obtained by comparing *m/z* values, retention times, and isotopic patterns, with confirmatory information from collisional cross-section values and MS/MS spectra. A score of analytical quality including intensity, peak shape, and retention time was used when a given metabolite was identified using more than one technique^27^. This annotation workflow led to a dataset of 278 identified metabolites. Unit variance scaling was applied to standardize the dataset and avoid any influence of intensity range on the observed differences. Principal component analysis (PCA) and orthogonal projections to latent structures-discriminant analysis (OPLS-DA) models with unit variance scaling were computed using SIMCA-P 15.0 software (Umetrics, Umea, Sweden). The supervised analysis of independent observations collected from different patients was carried out using standard OPLS-DA. ANOVA multiblock OPLS (AMOPLS) analysis was computed for the supervised analysis of paired observations involving repeated measurements. In that context, a multilevel strategy was implemented to account for this aspect, by separating within- from between-individuals sources of variability^28^. AMOPLS was computed after unit variance scaling as previously described under the MATLAB 8 environment (The MathWorks, Natick, USA)^29^. Random permutations of the response matrix were used to validate each model by simulating data under the null hypothesis (n = 1000). Models interpretation was based on scores to assess sample groupings and loadings to interpret directions of variation. Shared and Unique structure (SUS) plot was used to compare variable loadings between OPLS and AMOPLS models. Leave-one-out cross-validation was carried out to assess the predictive ability of the models (Q^2^).

Complementary univariate comparisons between preHD and postHD was carried out using paired t-test and the linear step-up procedure introduced by Benjamini and Hochberg was applied to estimate the Flase Discovery Rate for computing q-values^30^.

### Ethical statement

All patients included in this study provided informed consent. This study was approved by the local ethics committee (Commission cantonale d’éthique de la recherche (CCER), Geneva, Switzerland) and performed according to the Declaration of Helsinki.

## Results

We included 69 patients with CKD stage 3b or 4 (CKD group) and 35 on dialysis (HD group). As pre-sessional (preHD group) as well as post-sessional (postHD group) samples were drawn, 139 observations were obtained in total. Patients’ characteristics are described according to CKD versus HD group in Table 1. The median age of our population was 67.6 (58.1–75.0) years. Most patients were Caucasians (n = 82, 78.8%), males (n = 82, 78.8%) with cardiovascular risk factors such as diabetes (n = 51, 49.0%), hypertension (n = 89, 85.6%) and dyslipidaemia (n = 63, 60.6%). Characteristics were similar between CKD and HD patients, except for hypertension treatment, which was less prevalent in HD group. Median eGFR was 32.7 (18.3–46.8) mL/min/1.73m^2^ in CKD patients.

**Table 1:** Participants characteristics.

Characteristics	CKD (N = 69)	HD (N = 35)	P value
Age	67.1 (60.0–74.0)	70.0 (58.1–76.1)	0.55
Gender (Male)	57 (82.6%)	25 (71.4%)	0.21
Treated Dyslipidaemia	44 (63.8%)	19 (54.3%)	0.35
Treated Diabetes	31 (44.9%)	20 (57.1%)	0.30
Treated Hypertension	65 (94.2%)	24 (68.6%)	** < 0.001**
Body Mass Index	28.9 (24.5–32.9)	26.9 (22.1–30.8)	0.06

Continuous variables are presented as median (interquartile range) and non-parametric Mann–Whitney test was conducted for comparison. Categorical data are reported as numbers and proportions (%) and Fisher exact test was used for comparison. A threshold of *p* < 0.05 was considered statistically significant and indicated as bold value.

CKD, chronic kidney disease; HD, haemodialysis.

Sample collection was carried out under satisfactory conditions in terms of both quantity and quality, leading to a data matrix without any missing values or incomplete samples. The complete list of identified metabolites with descriptive statistics and biochemical information is provided as Supplementary Table S1.

### Unsupervised analysis (PCA)

**Figure 1:**
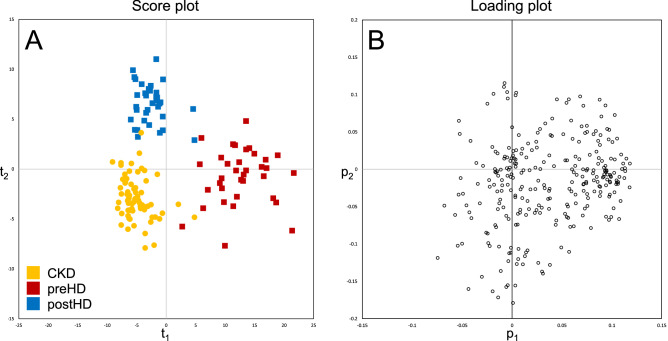
Unsupervised PCA results based on 278 identified metabolites (N = 139). (**A**): Score plot of individual patients. (**B**): Loading plot of individual metabolites. Abbreviations: PCA, principal component analysis; CKD, chronic kidney disease; preHD, before haemodialysis; postHD, after haemodialysis.

As a first step, PCA was carried out on all 139 samples to gain an overview of the collected data. On a patient level, the PCA score plot (Fig. 1A) showed clear clustering of the three patient’s groups (CKD, preHD and postHD) according to the first two principal components (t_1_ and t_2_). t_1_ explained 22.9% of the total variability, mainly highlighting metabolic patterns differentiating preHD from the two other groups. Moreover, CKD and postHD patients were clearly separated according to t_2_ explaining 7.3% of the total variability. The overall impact of considered clinical characteristics on the distribution of metabolic profiles was assessed using PCA and no significant trend was found (Supplementary Fig. S1).

The corresponding loading plot was investigated to assess metabolite contributions to the observed trends. Higher levels of most of the 278 identified metabolites were associated with large positive loading values on the first principal component (p_1_), thus illustrating a marked trend of accumulation in preHD samples compared to the two other groups (Fig. 1B). Moreover, smaller subsets of metabolites associated with large positive or negative loading values on the second principal component (p_2_) were highlighted. These compounds may constitute specific CKD or postHD patterns and further investigations were therefore carried out using supervised analysis to distinguish specific metabolic signatures associated with each group.

### Supervised analysis (OPLS-DA and AMOPLS)

**Figure 2:**
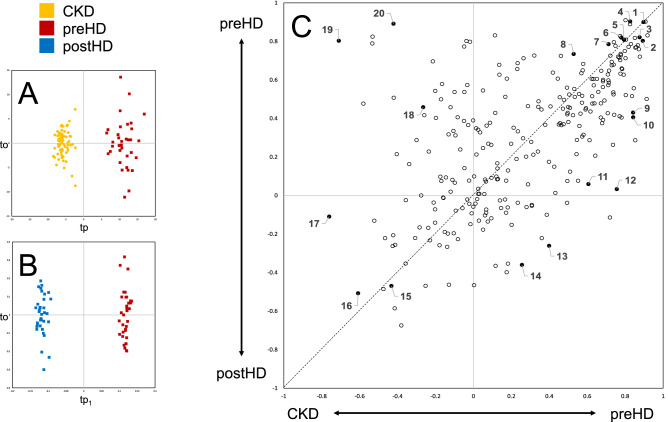
Supervised analysis results based on 278 identified metabolites. (**A**): OPLS-DA results (N = 104). (**B**): AMOPLS results (N = 70). (**C**): SUS plot (N = 139). (1) 5′-methylthioadenosine, (2) creatinine, (3) arabinose, (4) formylmethionine, (5) n-acetylmethionine, (6) myo-inositol, (7) N-acetylleucine, (8) 5-hydroxytryptophan, (9) indoxyl sulfate, (10) kynurenic acid, (11) 5a-DHT-17b-glucuronide, (12) cortisol 21-acetate, (13) 1-oleoyl-rac-glycerol, (14) gamma-linolenic acid, (15) biliverdin, (16) keto-isoleucin, (17) tryptophan, (18) guanidoacetic acid, (19) carnitine, (20) uric acid. Abbreviations: OPLS-DA, orthogonal projections to latent structures-discriminant analysis; AMOPLS, ANOVA multiblock OPLS; SUS, shared and unique structure; CKD, chronic kidney disease; preHD, before haemodialysis; postHD, after haemodialysis.

A first supervised model was evaluated to assess metabolic differences between CKD and preHD groups. Leave-one-out cross-validation showed the high prediction ability of the model (Q^2^ = 0.89). Accordingly, the two groups were clearly separated on the score plot (Fig. 2A).

The HD effect was then specifically investigated using the subset of 70 samples collected from 35 HD patients before (preHD) and after (postHD) dialysis. For that purpose, AMOPLS was implemented using a response design matrix involving (1) the *Patient* factor (35 levels), and (2) the *Haemodialysis* factor (2 levels). ANOVA decomposition revealed that inter-individual differences related to the *Patient* factor accounted for 57.8% of the total variability, metabolic alterations due to *Haemodialysis* for 22.2% and unexplained residuals for the remaining 20%. Permutation tests confirmed the validity of an optimal AMOPLS model with three latent variables (*p* < 0.015, *tp*_*1*_ predictive of *Haemodialysis*, *tp*_*2*_ predictive of *Patient* and *t*_*o*_ summarizing orthogonal variability), while the two main effects were found significant (*Patient* *p* < 0.001 and *Haemodialysis* *p* < 0.001). Samples collected from HD patients before (preHD) and after (postHD) dialysis were clearly separated on tp_1_ (Fig. 2B). Loadings associated with each metabolite with corresponding bootstrap 95% confidence intervals for the AMOPLS model are represented in descending order in Supplementary Fig. S2. As a complement, univariate comparisons between preHD and postHD were carried out and summarized using a Volcano plot in Supplementary Fig. S3.

Interpretation was then carried out by putting a specific focus on metabolites contributions using a SUS plot combining differences between (1) preHD and CKD groups as well as (2) preHD and postHD groups^28^. For that purpose, loading values from both models were scaled as correlation coefficients (i.e. *pcorr*) and combined on X-axis and Y-axis, respectively. As such, common metabolic trends could be clearly visualized, while specific metabolite behaviours were also highlighted (Fig. 2C). Metabolites with comparable loading pcorr values in both models exhibited similar variations across considered disease states (shared structure on the [(− 1;− 1), (+ 1; + 1)] diagonal),while metabolites with different trends varied specifically to a given situation (unique structure outside the diagonal). Coordinates of individual identified metabolites on the SUS plot are given in Supplementary Table S1.

### Individual metabolites description

**Figure 3:**
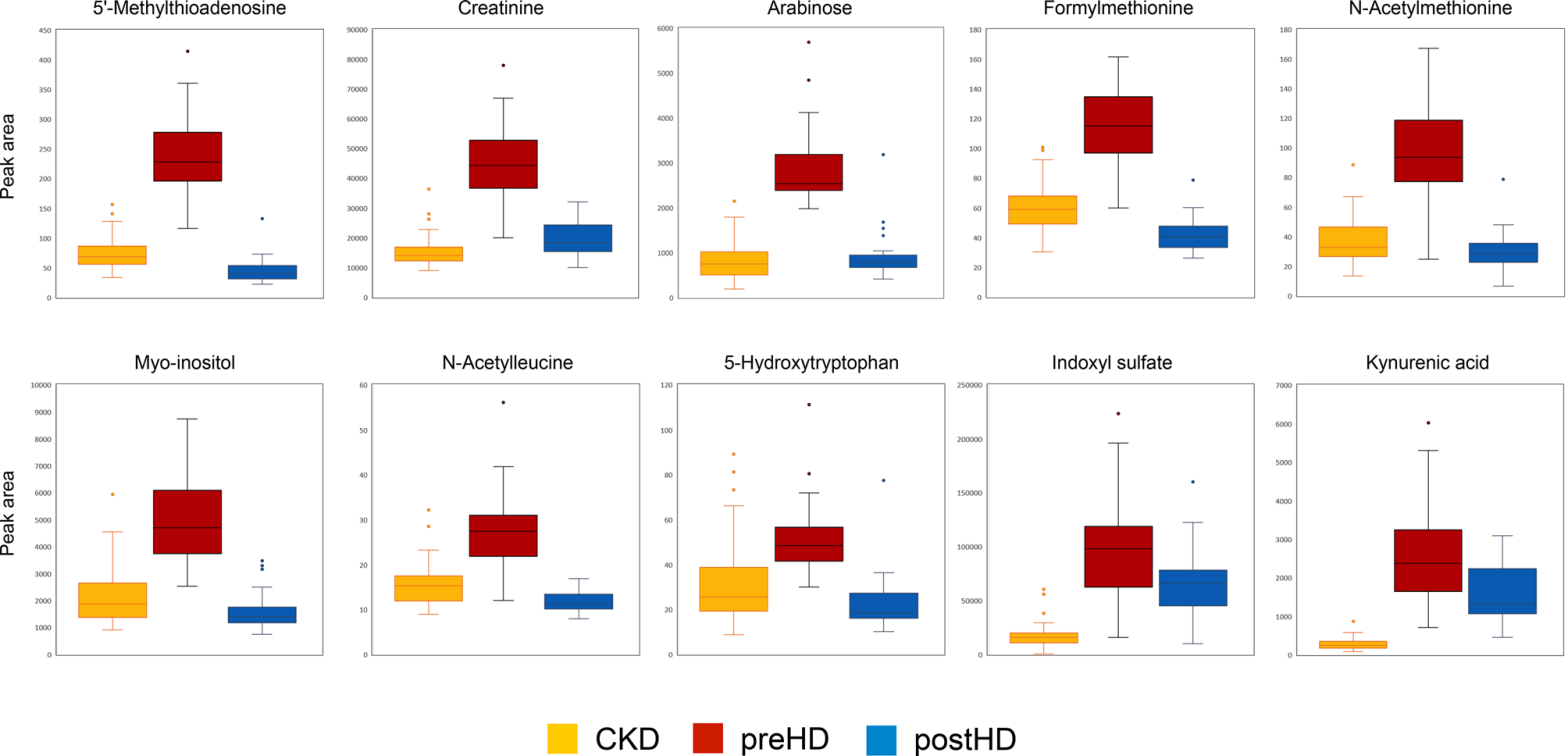
Box-plots of selected metabolites accumulating in preHD and significantly cleared by HD (N = 139). Abbreviations: preHD, before haemodialysis; CKD, chronic kidney disease; HD, haemodialysis; postHD, after haemodialysis.

**Figure 4:**
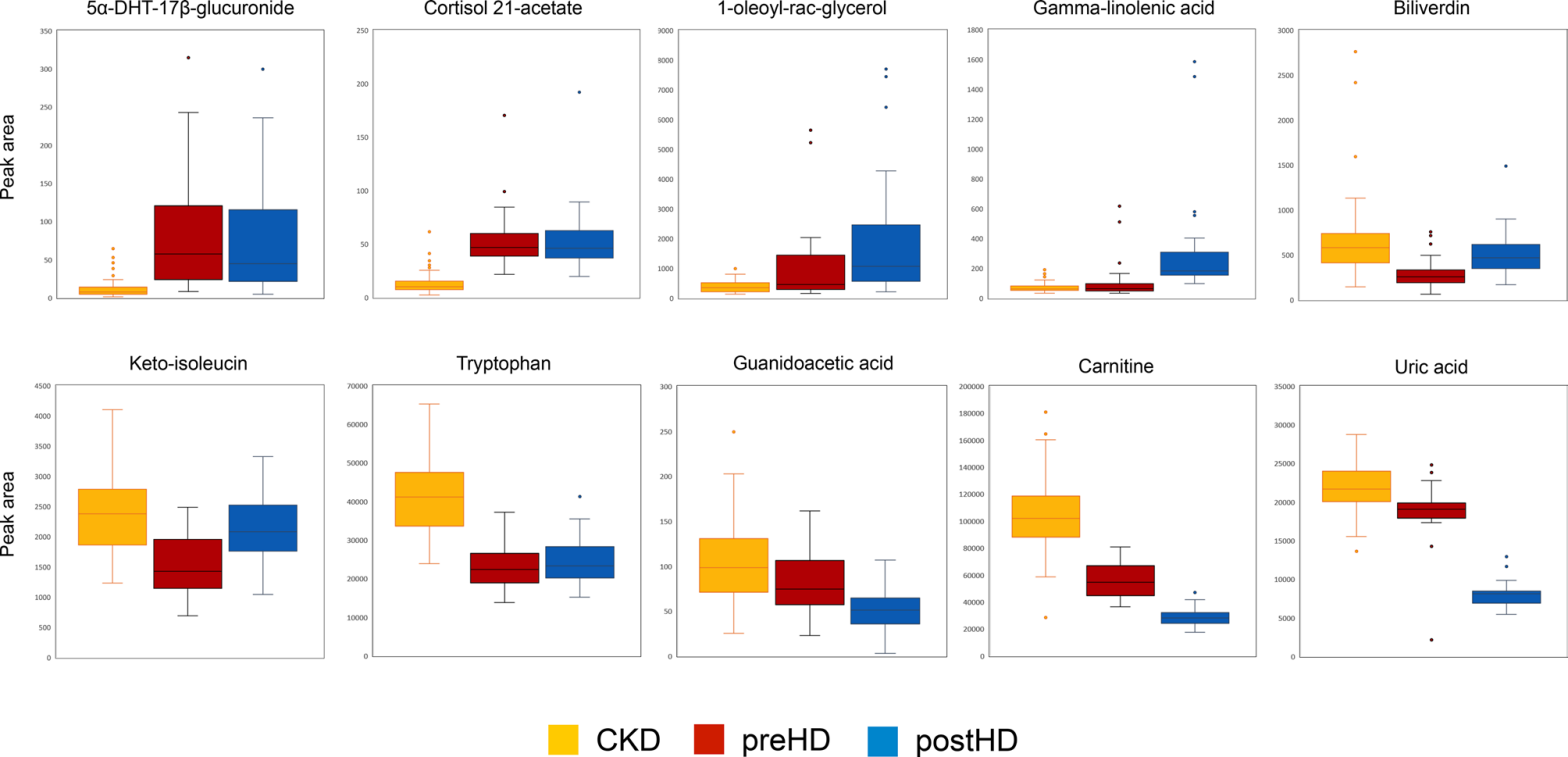
Box-plots of selected metabolites with distinct patterns regarding accumulation in preHD and clearance in HD (N = 139). Abbreviations: preHD, before haemodialysis; HD, haemodialysis; CKD, chronic kidney disease; postHD, after haemodialysis.

Investigation of specific metabolites was then carried out to go beyond the overall description of these global patterns. Specific metabolites were selected for illustrative purposes based on their position on the SUS plot (Fig. 2C) when considered clinically relevant. The latter offers a summary of differences observed between disease states (i.e. CKD, preHD and postHD). Selected metabolites accumulating in preHD compared to CKD and significantly cleared by HD are illustrated in Fig. 3. Selected metabolites with qualitatively distinct patterns regarding accumulation in preHD and clearance in HD are illustrated in Fig. 4.

## Discussion

In this study, an original metabolomics workflow combining different chromatographic separation modes was implemented to characterise metabolic profiles of CKD patients as well as the effect of HD on those profiles. Using such an approach, metabolic alterations effectively discriminating CKD stages and highlighting various effects of HD treatment were described among profiles composed of 278 identified metabolites.

Severity of CKD represented the main source of variability in our dataset (Fig. 1A), with preHD patients clearly standing out from CKD and postHD patients. These results suggest that HD treatment effectively allows patients to recover a metabolic profile closer to that of earlier stage of renal disease. However, a specific shift of the metabolic profile was also observed after dialytic therapy that could not be found in endogenous alteration of kidney function. A first overall assessment of individual metabolites contribution (Fig. 1B) revealed that no uniform shift in concentration could explain differences between CKD and preHD states. On the contrary, some metabolites were found to be increased in preHD as compared to CKD, while the opposite was true for other metabolites. Globally, differences could thus be readily observed in dialytic compared to endogenous clearance of various molecules.

In order to characterise biological patterns associated with specific disease states, supervised models were compared (Fig. 2C). Similar to uremic solutes considered in clinical practice, most identified metabolites increased in preHD compared to CKD and were efficiently cleared in postHD. While creatinine was obviously found in this group, other metabolites of interest with a similar behaviour could also be highlighted (Fig. 3). As such, N-acetyl amino-acids (e.g. *N*-acetylmethionine and *N*-acetylleucine) also belonged to this group. A correlation between renal function and some amino-acids has been previously reported and our findings would globally reinforce the hypothesis of *N*-acetylation as a detoxification mechanism in CKD^31^. Arabinose, 5′-methylthioadenosine, formylmethionine and myo-inositol have all previously been highlighted as uremic solutes^32^. Finally, 5-hydroxytryptophan, an amino-acid derived from tryptophan and a direct precursor of serotonin, also showed a similar metabolic profile across disease states. Globally, on a clinical point of view, metabolites presenting such a pattern could potentially represent toxic uremic solutes while also being candidate to provide assessment of kidney function or dialysis dose.

Several metabolites presented other specific profiles across disease states of clinical interest. First, while indoxyl sulfate and kynurenic acid displayed a metabolic profile qualitatively similar to creatinine, important quantitative differences were highlighted, as HD was not merely able to restore concentrations comparable to those of CKD patients (Fig. 3). This pattern would imply that patients requiring HD have significantly and persistently higher concentrations of those molecules compared to patients suffering from earlier stage CKD. Indoxyl sulfate is synthetized in the liver from indole, which is produced by the intestinal flora as a metabolite of tryptophan^33^. While being intrinsically a small molecular weight molecule, indoxyl sulfate is recognized as a protein-bound uremic toxin^34^. This metabolite has been reported to promote endothelial dysfunction by inducing oxidative stress in vitro^35^. It has also been shown to impair osteoblast function and promote abnormalities in bone turnover^36^. Indoxyl sulfate also experimentally induces the progression of glomerular sclerosis and renal failure^37^. Finally, this metabolite could play a role in the central nervous system dysfunction as it is thought to accumulate in the brain^38^. The sustained high levels of indoxyl sulfate in HD patients could thus participate to typical complications of this population such as cardiovascular events, bone mineral disease, decline of residual kidney function and cognitive impairment. Kynurenic acid, a product of tryptophan metabolism, has also been recognized as a protein-bound uremic toxin^34^. It is known to inhibit glutamatergic transmission in the mammalian brain and has neuroprotective and anticonvulsive properties in animal models^39^. However, increased levels of kynurenic acid could underlie cognitive decline in certain conditions as increased metabolism of this molecule has been described in Alzheimer’s disease, Down’s syndrome and Huntington disease^39^. Among HD patients, prevalence of cognitive impairment is extremely high and can reach 70%^40^. While microvascular disease is a major factor contributing to this phenomenon, it is also possible that retained uremic toxins contribute to this impairment, even in patients receiving adequate dialysis dose based on conventional urea kinetic model^40^. As with indoxyl sulfate, persistent elevated concentrations of kynurenic acid despite adequate HD prescription could contribute to this clinical burden.

Other metabolites displayed patterns of interest (Fig. 4). Guanidoacetic acid is synthetized in the proximal convoluted tubule of the kidney by transaminidation from arginine to glycine prior to conversion into creatine in the liver and its concentration is consequently reduced in CKD^41^. While highlighted as a component of human metabolism decades ago, guanidoacetic acid has regained attention in the past years as its supplementation was reported to favourably affect muscle health in rats as well as healthy volunteers and patients suffering from CKD^42,43^. We found that the concentration of guanidoacetic acid further decreased after dialysis thus contributing to profound depletion in patients requiring renal replacement therapy, a population where muscle wasting notably contributes to frailty and morbidity^44^. This highlights the non-selective dialytic removal of potentially useful biological compounds that may contribute to the high morbi-mortality of HD patients. Carnitine is crucial for energy production in tissues dependent on fatty acid oxidation such as cardiac and skeletal muscle^45^. While it accumulates in CKD due to decreased renal clearance, most HD patients exhibit carnitine deficiency as a result of non-selective dialytic losses^45^. Our results confirm a major impact of dialytic removal on carnitine levels as HD patients exhibited significantly lower concentrations than CKD patients, despite lower or abolished kidney function. Carnitine depletion in chronic HD patient was thus linked to adverse manifestations and regular supplementation is often advised^45^. As for carnitine, uric acid tends to accumulate in CKD to due reduced renal clearance but hyperuricemia is also thought to contribute to the development and progression of CKD^46^. Our findings indicate a similar profile for uric acid compared to carnitine with efficient dialytic clearance and decreased overall levels in HD compared to CKD patients. In contrast to carnitine metabolism, such a pattern could however prove beneficial in this case by mitigating the adverse consequences of elevated uric acid levels although evidences are sparse^46^. The first rate-limiting step of the kynurenine pathway is the production of kynurenine from tryptophan by the enzyme indoleamine dioxygenase 1 (IDO1)^47^. IDO1 activity has been found to be increased in CKD leading to decreased levels of tryptophan in renal disease^48^. As the kynurenine pathway is linked to systemic inflammation, a recent study found that reduced levels of tryptophan associated with incident cardiovascular disease in CKD patients even when adjusting for traditional risk factors^47^. Moreover, it has been postulated that lower levels of tryptophan in CKD patients would decrease melatonin synthesis thus offering a plausible explanation to the poorly understood CKD-associated fatigue phenomenon^49^. In our study, we show that tryptophan levels, while not lowered by dialysis itself, are further reduced in HD compared to CKD patients thus potentially contributing to explain the high cardiovascular risk of HD patients as well as the efficiency of melatonin to improve sleep disturbances in this population^50^. Low protein diet combined with ketoacid analogues has been advocated to preserve renal function and reduce uremic toxins production in CKD patients^51^. We show that keto-isoleucin, a typical compound of such supplements, is not depleted by dialysis but is still found in significantly lower amount in HD compared to CKD patients. Thus, while evidences are currently lacking, this would support an interesting role for ketoacid supplementation in HD patients. As a group, steroid glucuronides (e.g. 5α-DHT-17β-glucuronide) showed a distinctive pattern as they dramatically increased with decline in renal function but were not cleared by dialysis. This is in agreement with an accumulation of phase 2 glucuronidated metabolites in renal failure^52^. Other steroid compounds (e.g. cortisol 21-acetate) showed a similar profile. From a clinical standpoint, although inter-individual variation should be first characterized, metabolites with such a profile would constitute ideal candidates to estimated residual kidney function while on renal replacement therapy.

Based on our results, hypotheses regarding the impact of metabolites physicochemical properties on dialytic removal can be formulated. As expected, molecules with low molecular weight (< 300 Da) and high water solubility (> 1 g/L) (e.g. 5′-methylthioadenosine, creatinine, arabinose, formylmethionine, N-acetylmethionine, myo-inositol, N-acetylleucine, 5-hydroxytryptophan, carnitine and uric acid) were efficiently removed by HD. Molecules with similar molecular weight but lower water solubility (< 1 g/L) and negatively charged (e.g. indoxyl sulphate and kynurenic acid) showed decreased removal during HD, potentially owing to higher volume of distribution and repulsive interaction with the negatively charged HD membrane respectively^53^. The steroid 5α-DHT-17β-glucuronide, characterized by a relatively higher molecular weight (> 450 Da), lower water solubility (< 0.5 g/L) and a negative charge was virtually not removed by HD. Unlike other amino acids, tryptophan is largely protein-bound in serum thus preventing efficient removal by HD, as seen in our study as well as previous reports^5,54^. Finally, concentration of some metabolites increased after HD. Theoretically, ultrafiltration-induced haemoconcentration or intradialytic endogenous release could explain such a finding. As HD has long been recognized as a catabolic process, it is plausible that per-dialytic levels of metabolites such as 1-oleoyl-rac-glycerol and keto-isoleucine increased as a result of activated catabolic pathways of lipids and amino-acids respectively^5,13,55^. However, reliable differentiation and quantification between endogenous metabolism and extracorporeal clearance was not possible in our study as it would require controlled clinical conditions as well as profiling of spent dialysates^5^.

Our study differs from previous publications in the field. While some reports included patients requiring HD, several provided preHD analysis only^22,34^. While other considered preHD as well as postHD samples, healthy control participants were usually included for comparison purposes^5,18,20,21^. We, on the other hand, have aimed at describing global metabolic alterations in patients requiring HD as compared to CKD patients not requiring renal replacement therapy in order to corroborate distinct observed clinical trajectories.

Our study has limitations. While, we were able to characterise metabolic alterations in a broad range of CKD severity, no patient was included with an eGFR > 45 mL/min and we could not identify early markers of kidney dysfunction. Moreover, being cross-sectional in nature, we were able to describe metabolic patterns associated with certain disease states. However, whether those patterns are linked to CKD progression remained unanswered and will need further prospective studies.

## Conclusion

In this cross-sectional study, a novel metabolomics framework combining RPLC and HILIC chromatographic separation modes was implemented to offer broad metabolome coverage in CKD. This methodology generated extended metabolic profiles that could readily discriminate different CKD stages and highlight the effect of HD based on 278 identified metabolites. Global differences in metabolic profile were described between patients requiring HD and those with earlier stage of CKD. Moreover, individual molecules displayed various patterns in regards to renal and dialytic clearances. Such differences could contribute to explain clinical discrepancies between patients requiring HD and those with relatively preserved kidney function.

## Supplementary information

Supplementary Information 1.

Supplementary Information 2.

## Data Availability

Data supporting the findings of this study are available from the corresponding author upon reasonable request.

## References

[1] Hocher B, Adamski J (2017). Metabolomics for clinical use and research in chronic kidney disease. Nat. Rev. Nephrol..

[2] Kalim S, Rhee EP (2017). An overview of renal metabolomics. Kidney Int..

[3] Boelaert J (2017). Metabolic profiling of human plasma and urine in chronic kidney disease by hydrophilic interaction liquid chromatography coupled with time-of-flight mass spectrometry: A pilot study. Anal. Bioanal. Chem..

[4] Gagnebin Y, Julien B, Belén P, Serge R (2018). Metabolomics in chronic kidney disease: Strategies for extended metabolome coverage. J. Pharm. Biomed. Anal..

[5] Rhee EP (2010). Metabolite profiling identifies markers of uremia. J. Am. Soc. Nephrol..

[6] Shah VO (2013). Plasma metabolomic profiles in different stages of CKD. Clin. J. Am. Soc. Nephrol..

[7] Ivanisevic J (2013). Toward ’Omic scale metabolite profiling: A dual separation-mass spectrometry approach for coverage of lipid and central carbon metabolism. Anal. Chem..

[8] Spagou K (2011). HILIC-UPLC-MS for exploratory urinary metabolic profiling in toxicological studies. Anal. Chem..

[9] Hill NR (2016). Global prevalence of chronic kidney disease - A systematic review and meta-analysis. PLoS ONE.

[10] Tonelli M (2006). Chronic kidney disease and mortality risk: A systematic review. J. Am. Soc. Nephrol..

[11] Vanholder R (2003). Review on uremic toxins: Classification, concentration, and interindividual variability. Kidney Int..

[12] Garibotto Giacomo G (2010). Amino acid and protein metabolism in the human kidney and in patients with chronic kidney disease. Clin. Nutr..

[13] Lim VS, Kopple JD (2000). Protein metabolism in patients with chronic renal failure: Role of uremia and dialysis. Kidney Int..

[14] Vaziri ND (2006). Dyslipidemia of chronic renal failure: The nature, mechanisms, and potential consequences. Am. J. Physiol. Ren. Physiol..

[15] Couser WG, Remuzzi G, Mendis S, Tonelli M (2011). The contribution of chronic kidney disease to the global burden of major noncommunicable diseases. Kidney Int..

[16] Neovius M, Jacobson SH, Eriksson JK, Elinder CG, Hylander B (2014). Mortality in chronic kidney disease and renal replacement therapy: A population-based cohort study. BMJ Open.

[17] Noordzij M, Jager KJ (2014). Increased mortality early after dialysis initiation: A universal phenomenon. Kidney Int..

[18] Sato E (2011). Metabolomic analysis of human plasma from haemodialysis patients. Eur. J. Clin. Invest..

[19] Duranton F (2014). Plasma and urinary amino acid metabolomic profiling in patients with different levels of kidney function. Clin. J. Am. Soc. Nephrol..

[20] Kromke M (2016). Profiling human blood serum metabolites by nuclear magnetic resonance spectroscopy: A comprehensive tool for the evaluation of hemodialysis efficiency. Transl. Res..

[21] Zhang ZH (2017). Removal of uremic retention products by hemodialysis is coupled with indiscriminate loss of vital metabolites. Clin. Biochem..

[22] Chen Y, Wen P, Yang J, Niu J (2020). Plasma metabolomics profiling in maintenance hemodialysis patients based on liquid chromatography quadrupole time-of-flight mass spectrometry. Kidney Dis..

[23] Gagnebin Y (2019). Toward a better understanding of chronic kidney disease with complementary chromatographic methods hyphenated with mass spectrometry for improved polar metabolome coverage. J Chromatogr. B Anal. Technol. Biomed. Life Sci..

[24] Levey AS (2009). A new equation to estimate glomerular filtration rate. Ann. Intern. Med..

[25] González-Ruiz V (2017). Unravelling the effects of multiple experimental factors in metabolomics, analysis of human neural cells with hydrophilic interaction liquid chromatography hyphenated to high resolution mass spectrometry. J. Chromatogr. A.

[26] Blaženović I, Kind T, Ji J, Fiehn O (2018). Software tools and approaches for compound identification of LC-MS/MS data in metabolomics. Metabolites.

[27] Pezzatti J (2019). A scoring approach for multi-platform acquisition in metabolomics. J. Chromatogr. A.

[28] Wiklund S (2008). Visualization of GC/TOF-MS-based metabolomics data for identification of biochemically interesting compounds using OPLS class models. Anal. Chem..

[29] Boccard J, Rudaz S (2016). Exploring Omics data from designed experiments using analysis of variance multiblock Orthogonal Partial Least Squares. Anal. Chim. Acta.

[30] Benjamini Y, Hochberg Y (1995). Controlling the false discovery rate: A practical and powerful approach to multiple testing. J. R. Stat. Soc. Ser. B.

[31] Sekula P (2016). A metabolome-wide association study of kidney function and disease in the general population. J. Am. Soc. Nephrol..

[32] Tanaka H, Sirich TL, Plummer NS, Weaver DS, Meyer TW (2015). An enlarged profile of uremic solutes. PLoS ONE.

[33] Brunet P, Dou L, Cerini C, Berland Y (2003). Protein-bound uremic retention solutes. Adv. Ren. Replace. Ther..

[34] Duranton F (2012). Normal and pathologic concentrations of uremic toxins. J. Am. Soc. Nephrol..

[35] Dou L (2007). The uremic solute indoxyl sulfate induces oxidative stress in endothelial cells. J. Thromb. Haemost..

[36] Iwasaki Y (2006). Uremic toxin and bone metabolism. J. Bone Miner. Metab..

[37] Niwa T, Ise M, Miyazaki T (1994). Progression of glomerular sclerosis in experimental uremic rats by administration of indole, a precursor of indoxyl sulfate. Am. J. Nephrol..

[38] Ohtsuki S (2002). Role of blood-brain barrier organic anion transporter 3 (OAT3) in the efflux of indoxyl sulfate, a uremic toxin: Its involvement in neurotransmitter metabolite clearance from the brain. J. Neurochem..

[39] Kepplinger B (2005). Age-related increase of kynurenic acid in human cerebrospinal fluid—IgG and β2-microglobulin changes. Neurosignals.

[40] Seliger SL, Weiner DE (2013). Cognitive impairment in dialysis patients: Focus on the blood vessels?. Am. J. Kidney Dis..

[41] Levillain O, Marescau B, De Deyn PP (1995). Guanidino compound metabolism in rats subjected to 20% to 90% nephrectomy. Kidney Int..

[42] Tsubakihara Y, Hayashi T, Shoji T (2012). Effects of guanidinoacetic acid(gaa) supplementation in rats with chronic renal failure(crf). Kidney Res. Clin. Pract..

[43] Ostojic SM (2015). Advanced physiological roles of guanidinoacetic acid. Eur. J. Nutr..

[44] Hanna RM, Ghobry L, Wassef O, Rhee CM, Kalantar-Zadeh K (2020). A practical approach to nutrition, protein-energy wasting, sarcopenia, and cachexia in patients with chronic kidney disease. Blood Purif..

[45] Ahmad S (2001). L-Carnitine in dialysis patients. Semin. Dial..

[46] Jalal DI, Chonchol M, Chen W, Targher G (2013). Uric acid as a target of therapy in CKD. Am. J. Kidney Dis..

[47] Konje VC (2020). Tryptophan levels associate with incident cardiovascular disease in chronic kidney disease. Clin. Kidney J..

[48] Bao YS (2013). Serum levels and activity of indoleamine2,3-dioxygenase and tryptophanyl-tRNA synthetase and their association with disease severity in patients with chronic kidney disease. Biomarkers.

[49] Luft FC (2014). The author replies. Kidney Int..

[50] Koch BCP (2009). The effects of melatonin on sleep-wake rhythm of daytime haemodialysis patients: A randomized, placebo-controlled, cross-over study (EMSCAP study). Br. J. Clin. Pharmacol..

[51] Koppe, L., De Oliveira, M. C. & Fouque, D. Ketoacid analogues supplementation in chronic kidney disease and future perspectives. *Nutrients* **11**, (2019). 10.3390/nu11092071 PMC6770434 31484354

[52] Ng DPK (2012). A metabolomic study of low estimated GFR in non-proteinuric type 2 diabetes mellitus. Diabetologia.

[53] Clark WR, Gao D, Neri M, Ronco C (2017). Solute transport in hemodialysis: Advances and limitations of current membrane technology. Contrib. Nephrol..

[54] Walser M, Hill SB (1993). Free and protein-bound tryptophan in serum of untreated patients with chronic renal failure. Kidney Int..

[55] Holecek M, Siman P, Vodenicarovova M, Kandar R (2016). Alterations in protein and amino acid metabolism in rats fed a branched-chain amino acid- or leucine-enriched diet during postprandial and postabsorptive states. Nutr. Metab..

