# Supplementary material for: Exploring blood alterations in chronic kidney disease and haemodialysis using metabolomics
Source: Sci Rep. 2020 Nov 11;10:19502. doi: 10.1038/s41598-020-76524-1 (PMC7658362; doi:10.1038/s41598-020-76524-1)
Supplement: Supplementary file 1 — Supplementary Information 1. [file 41598_2020_76524_MOESM1_ESM.pdf]

EXPLORING BLOOD ALTERATIONS IN CHRONIC KIDNEY DISEASE AND HAEMODIALYSIS USING METABOLOMICS.

Yoric GAGNEBIN; David A. JAQUES; Serge RUDAZ; Sophie de SEIGNEUX; Julien BOCCARD; Belén PONTE.

SUPPLEMENTARY MATERIAL

Supplementary table S1: Biochemical characteristics of the 278 identified metabolites.

Supplementary figure S1: Score plots of unsupervised PCA results based on 278 identified metabolites according to clinical characteristics (N=139).

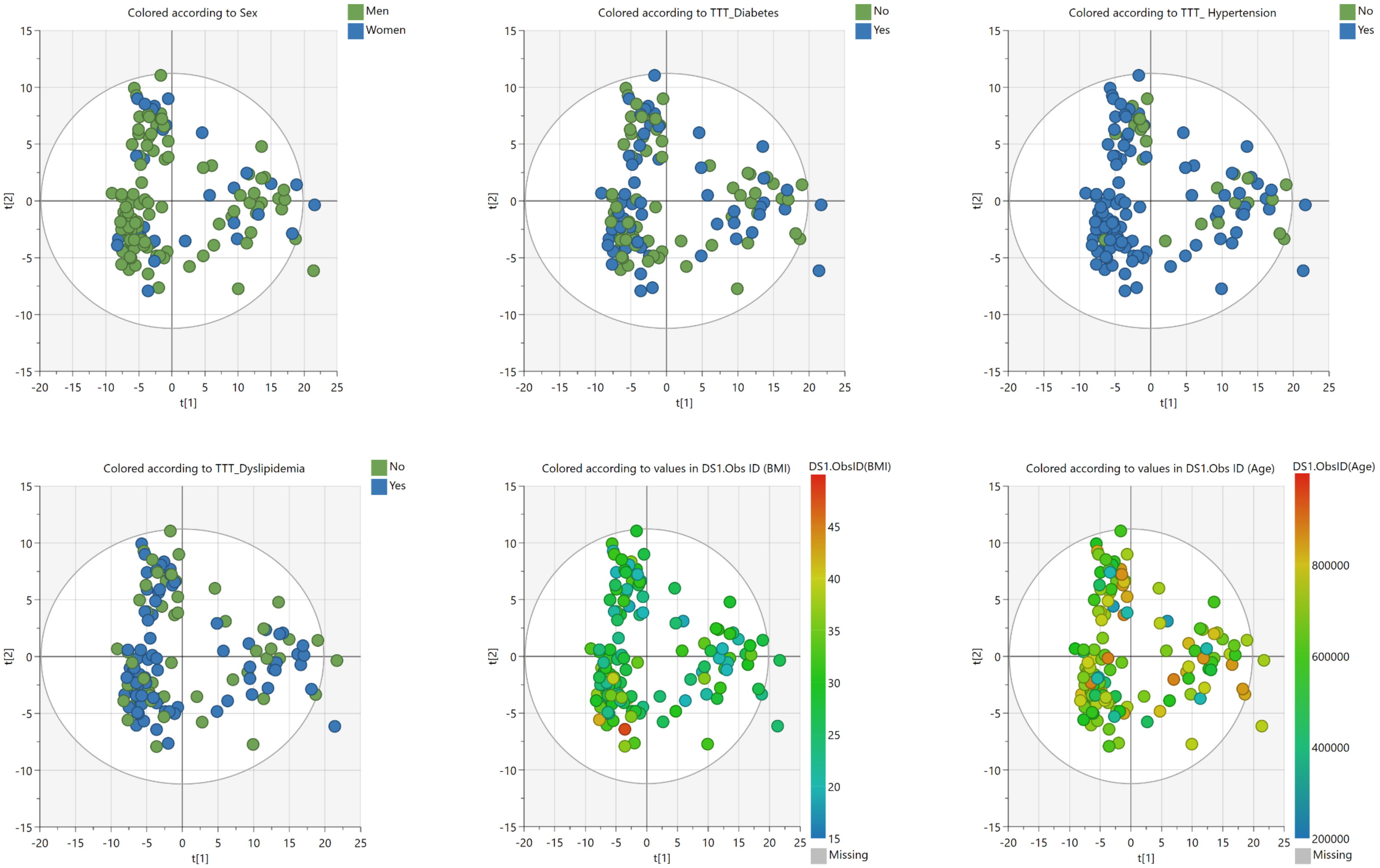

Supplementary figure S2: Loading plot of 278 identified metabolites in AMOPLS with associated 95% bootstrap confidence intervals (N=70).

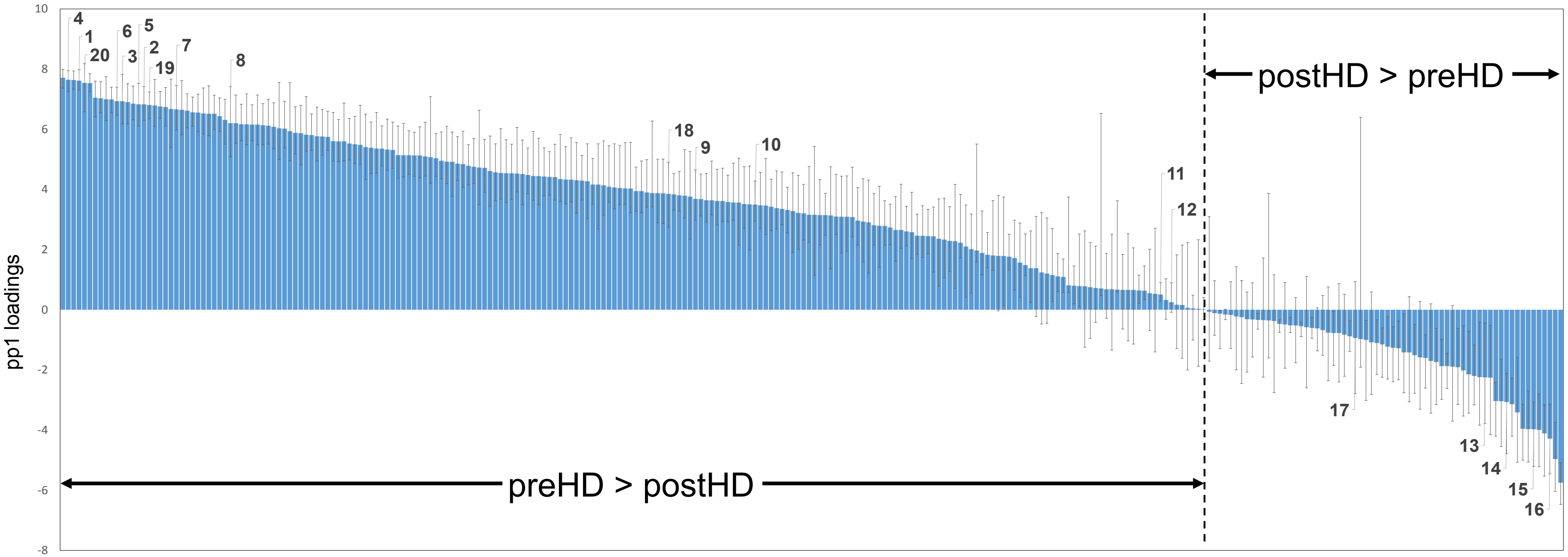

(1) 5'-methylthioadenosine, (2) creatinine, (3) arabinose, (4) formylmethionine, (5) n-acetylmethionine, (6) myo-inositol, (7) N-acetylleucine, (8) 5-hydroxytryptophan, (9) indoxyl sulfate, (10) kynurenic acid, (11) 5a-DHT-17b-glucuronide, (12) cortisol 21-acetate, (13) 1-oleoyl-rac-glycerol, (14) gamma-linolenic acid, (15) biliverdin, (16) keto-isoleucin, (17) tryptophan, (18) guanidoacetic acid, (19) carnitine, (20) uric acid.

Abbreviations: AMOPLS, ANOVA multiblock OPLS; OPLS, orthogonal projections to latent structures; preHD, before haemodialysis; postHD, after haemodialysis.

Supplementary figure S3: Volcano plot of statistical significance against fold-change between preHD and postHD (N=70).

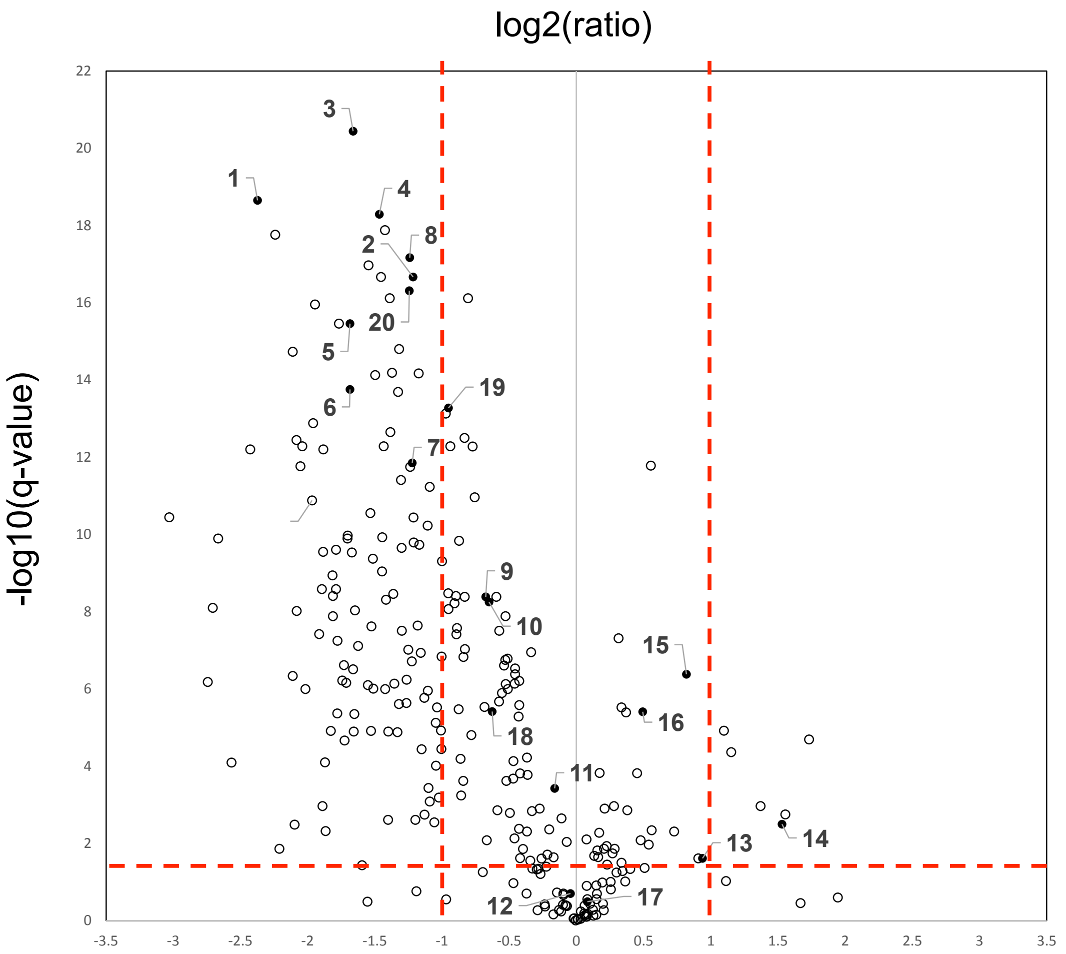

A paired t-test with False Discovery Rate correction was carried out for computing q-values. A fold change threshold of 2 and a q-value limit of 0.05 are displayed as red dashed lines.
